# Biology and utilization of R2 retrotransposons

**DOI:** 10.1080/15476286.2025.2521890

**Published:** 2025-06-25

**Authors:** Shengqiu Luo, Qicheng Chen, Yangcan Chen, Wei Li

**Affiliations:** a State Key Laboratory of Organ Regeneration and Reconstruction, Institute of Zoology, Chinese Academy of Sciences, Beijing, China; b University of Chinese Academy of Sciences, Beijing, China; c Beijing Institute for Stem Cell and Regenerative Medicine, Beijing, China

**Keywords:** R2 retrotransposons, protein domains, RNA, ribozyme, mechanism, reverse transcription, gene integration, genome engineering

## Abstract

R2 elements serve as a class of non-long terminal repeat (non-LTR) retrotransposons found in animal genomes that specifically insert into the ribosomal DNA (rDNA) sequences of host genomes. Each R2 element contains a single open reading frame (ORF) encoding a multifunctional protein with nucleic acid-binding, reverse transcriptase, and endonuclease activities, enabling specific genomic integration via a mechanism called target-primed reverse transcription (TPRT). As a classical model for studying retrotransposition mechanisms, R2 elements possess unique biological properties and precise integration capabilities, which have inspired novel genome engineering strategies. In this review, we summarize the components and integration mechanisms of R2 retrotransposons and highlight the recent advances in employing these mobile elements for targeted gene integration. Finally, we present future directions for the utilization of R2 retrotransposons as novel biotechnological tools.

## Introduction

R2 elements belong to a distinct class of mobile elements and specifically integrate into the ribosomal DNA (rDNA) locus. R2 sequences were first identified as insertion sequences within the 28S rDNA genes of *Drosophila melanogaster* by Dawid et al. [1] and Roiha et al. [2] in 1981 and were later detected in other insect genomes, such as *Calliphora erythrocephala* [3] and *Bombyx mori* [4]. Subsequent studies revealed that R2-encoded proteins contain reverse transcriptase (RT) domains homologous to those of non-long terminal repeat (non-LTR) retrotransposons [5], suggesting that R2 elements represent a new class of non-LTR retrotransposons. To date, R2 elements have been identified in insects, fish, birds, and other taxa [6–9], but not in mammals.

**Figure 1.**
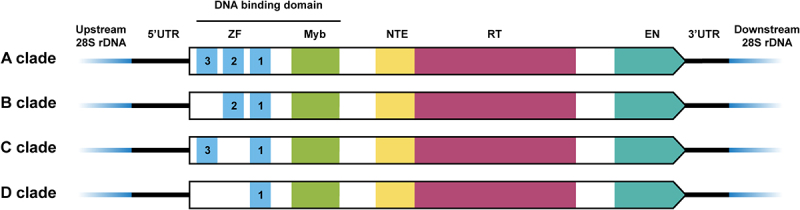
Components of R2 retrotransposons from clades A to D.

Each R2 element consists of a single open reading frame (ORF) flanked by variable-length untranslated regions (5’ UTR and 3’ UTR) (Figure 1). The ORF encodes the R2 protein, which contains several functional domains, including an N-terminal DNA-binding domain, a C-terminal endonuclease (EN) domain, and a centrally located reverse transcriptase domain (Figure 1). R2 elements replicate through a mechanism known as target-primed reverse transcription (TPRT), in which R2 proteins specifically bind to RNA transcripts that also serve as translation templates, nick the first strand of the DNA target site, and use the 3’ end of the exposed DNA to initiate reverse transcription with their own RNAs as templates [10,11].

Each R2 element consists of an ORF flanked by variable-length untranslated regions. R2 retrotransposons branch into four subclades, from A to D. UTR, untranslated regions; ZF, zinc finger; Myb, Myb-like motif; NTE, N-terminal extension; RT, reverse transcriptase domain; EN, endonuclease.

A key characteristic of R2 elements is their integration specificity. Most R2 elements target a fixed insertion site within 28S rDNA genes of the host genome. Notably, this site remains conserved in the genome of most eukaryotic species, underscoring its potential as a gene engineering tool for precise gene integration in diverse species [12]. In this review, we first outline the current understanding of the components and integration mechanisms of R2 retrotransposons. We then focus on the recent advances in employing R2 retrotransposons as biotechnological tools for gene addition. Finally, we highlight future directions for the utilization of R2 retrotransposons in tool development.

## Components of R2 elements

### Protein component

#### N-terminal DNA-binding domain

The N-terminal region of the R2 protein contains a DNA-binding domain composed of zinc finger (ZF) and Myb-like motifs, which binds to the specific sequence near the rDNA insertion site and is crucial for the site-specific recognition of R2 retrotransposons [13,14]. Some R2 family members, such as R9Av (from *Adineta vaga*), target different rDNA sites, a difference possibly due to the distinctive N-terminal domain [15,16]. Although rDNA sequences at the R2-insertion sites are nearly identical across eukaryotes [12], the number of ZF motifs in R2 varies. Based on the different numbers of ZFs and evolutionary analysis, R2 retrotransposons are classified into four subclades (A, B, C, and D) [7]. The R2-A clade contains three ZF motifs (ZF1, ZF2, and ZF3); the R2-B clade contains ZF1 and ZF2; the R2-C clade contains ZF1 and ZF3; and the R2-D clade contains only ZF1, which is the closest to the RT domain (Figure 1). A recent study has suggested that the cross-clade conserved ZF1-Myb is essential for the insertion of transgenes into rDNA by A-clade R2 proteins, but the absence of ZF2 and ZF3 reduces the insertion activity and the accuracy of the 5’ junction formation [17].

#### Reverse transcriptase domain

RTs synthesize DNA from RNA templates and are found in various genomic elements (e.g. group II introns, telomerases, retroviruses, and retrotransposons) [18]. R2-RT is more similar to the RTs of group II introns and telomerases than to those of retroviruses and LTR retrotransposons [19,20]. Compared with other RTs encoded by LTR retrotransposons, R2-RT exhibits distinct properties. R2-RT initiates reverse transcription using the exposed 3’ hydroxyl group of DNA [21] and can jump from one RNA template to another to perform continuous cDNA synthesis on non-continuous templates, a process called “template jumping” [22,23]. This process does not require sequence similarity between two RNA templates [23]. In addition, R2-RT lacks the RNase H domain and the corresponding activity to degrade RNA templates [24]. However, *in vitro* experiments indicate that R2-RTs can remove RNA templates by displacing RNA strands annealed to ssDNA templates [24]. R2-RT contains two N-terminal extension domains (NTE0 and NTE-1) [25]. The NTE-1 part is involved in 3’ UTR RNA binding and may participate in template switching during TPRT [25–28].

#### Endonuclease domain

The C-terminal region of the R2 ORF contains an endonuclease domain. Based on the structural and phylogenetic features of endonucleases, non-LTR retrotransposons can be divided into restriction-like endonuclease-encoding and apurinic-apyrimidinic endonuclease-encoding types. The former subclass to which R2-EN belongs represents an ancient lineage in evolutionary analysis [29]. Many members of this subclass have site-specific integration profiles [30]. R2-EN nicks the first strand of the target DNA and exposes its 3’ end to initiate TPRT. Recent studies have shown that R2-EN also contributes to the integration specificity of R2 retrotransposons [26].

### RNA component

#### 3’ UTR

The 3’ UTR in R2 RNA is essential for specific recognition and reverse transcription by the R2 protein [21]. Given the low sequence conservation of the 3’ UTR of R2 RNAs across different species, this specific recognition is thought to depend primarily on the secondary structure of the R2 3’ UTR [31,32]. This is further supported by experiments in which non-core stem-loop structures were removed from the 3’ UTR, and the remaining sequences retained or even enhanced the efficiency of R2 retrotransposition [12,26,28].

During TPRT, cDNA synthesis does not require sequence similarity between the 3’ UTR and the downstream 28S rDNA sequence [21]. However, experimental results indicate that adding homologous arm sequences upstream and downstream of the insertion site at the 5’ and 3’ ends of R2 RNA facilitates R2 integration [12,33–35]. Additionally, recent studies on R2Bm have shown that the 3’ UTR also possesses a template positioning function, which influences TPRT-primed full-length cDNA synthesis [28].

#### 5’ UTR

The secondary structure of the 5’ UTR is more complex than that of the 3’ UTR. The 5’ UTR together with the upstream rRNA sequence can form a hepatitis delta virus (HDV)-like ribozyme that undergoes self-cleavage [12,36–38]. Multiple sequence alignments indicate that this HDV-like ribozyme structure is conserved across R2 elements [12]. R2 RNA is co-transcribed with rRNA and matures via ribozyme self-cleavage [36], suggesting a 5’ cap-independent mechanism for translation initiation [39]. In the absence of a detectable R2-specific promoter, the HDV-like ribozyme in the R2 retrotransposons is considered to promote translation initiation [37]. The 5’ UTR also contributes to R2 retrotransposition. Ribozymes with reduced or abolished self-cleavage activity compromise R2 gene insertion efficiency in human cells; similarly, truncations in the remaining sequences impair insertion efficiency [12]. A recent study has demonstrated that the structural stability of the HDV-like ribozyme module enhances insertion efficiency by protecting processed R2 transcripts from 5’ exonucleolytic degradation [38].

#### 5’ RNA

The 5’ RNA is an RNA segment at the 5’ terminus of the R2 ORF and is located downstream of the 5’ UTR. Although 5’ RNA exists in some R2 retrotransposons from insects, such as R2Bm (from *Bombyx mori*), whether this motif is conserved in other R2 retrotransposons still needs to be explored [40]. The secondary structure of 5’ RNA includes a conserved pseudoknot, which suggests that this structure may be important for translation initiation [41]. 5’ RNA has been reported to participate in the regulation of the integration process [40,42]. A recent structural analysis of R2Bm provides molecular insights into the regulatory mechanism: when the R2 protein binds to the R2 3’ UTR, the Claw 3 domain of the 5’ RNA dissociates from the R2 protein due to competition from the 3’ UTR, thereby inhibiting the cleavage of the second strand [27].

## Integration of R2 retrotransposons

The integration of the R2 retrotransposon involves multiple sequential steps. Given that almost all *in vitro* biochemical experiments utilize the R2Bm protein as a model, the subsequent content will focus primarily on the integration mechanism of R2Bm retrotransposons.

**Figure 2.**
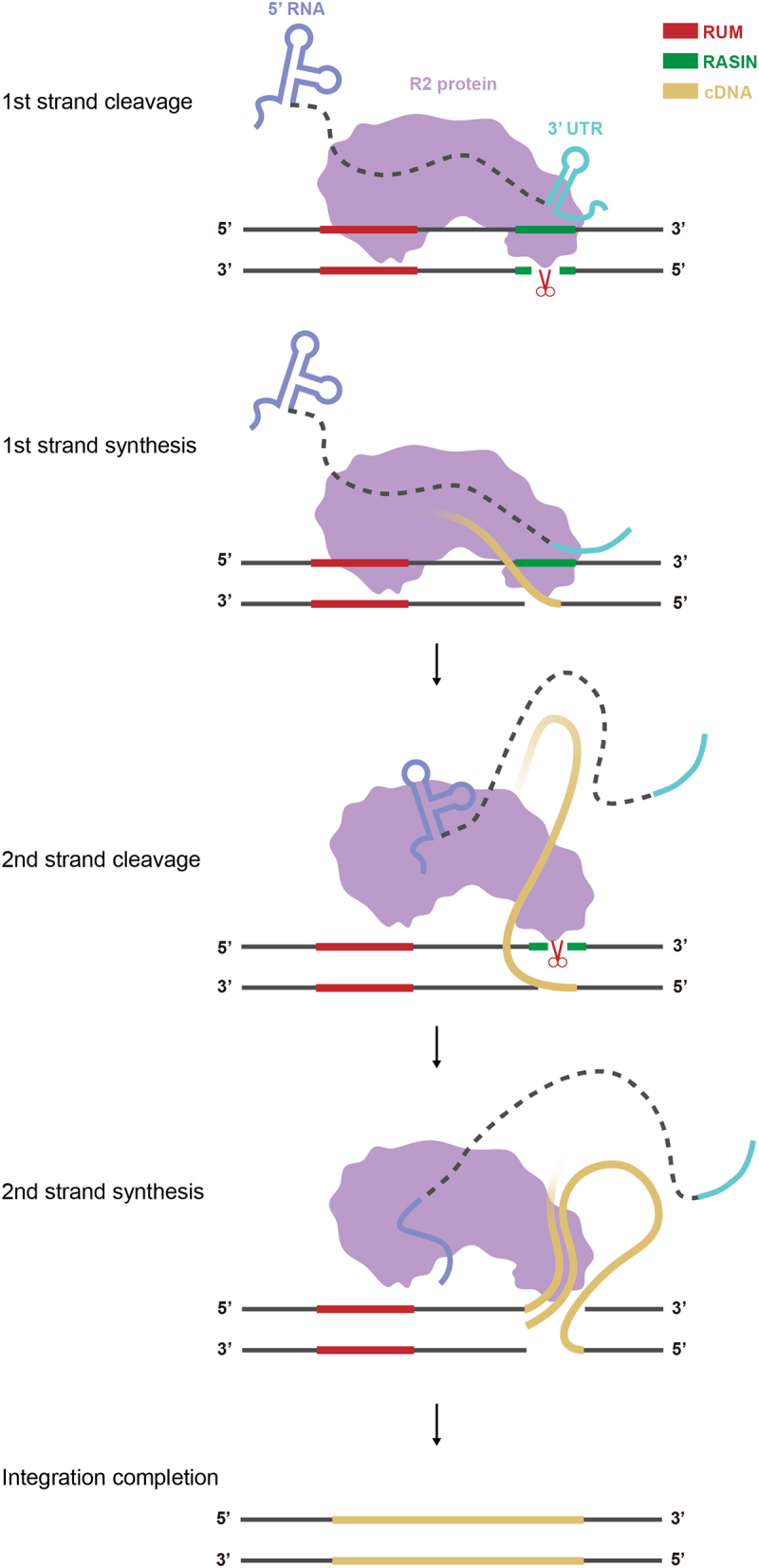
Schematic of R2 integration model.

Before integration begins, NTE-1 of the RT domain participates in recognizing and binding to the 3’ UTR of R2 RNA [25], forming a ribonucleoprotein (RNP) complex. The RNP complex specifically recognizes rDNA sequences that contain two conserved DNA motifs: the retrotransposon upstream motif (RUM) and retrotransposon-associated insertion site (RASIN) [26], which are alternatively referred to as the DNA-recognition region (Drr) and DNA-cleavage region (Dcr) [27], respectively. The RUM motif is cooperatively recognized by the zinc finger (ZF) domain, Myb-like domain, and loop 6a of the RT domain in the R2Bm protein. The RASIN motif undergoes unwinding near the RLE domain of the R2 protein in the RNP, determining both the nicking position and the retrotransposon insertion site. Following first-strand cleavage by the RLE domain at the unwound site, reverse transcriptase initiates first-strand cDNA synthesis using the exposed DNA 3’ end [10]. This critical mechanism is termed TPRT (Figure 2). Structural evidence demonstrated that sequence variations in the spacer between the RUM and RASIN motifs had minimal impact on R2 cleavage activity, while the tolerance for spacer length was very low. Any increase or decrease in spacer length significantly reduces the activity of the R2 protein [26,27].

The current model of R2 integration is shown. First, the complex formed by R2 protein and R2 RNA recognizes the RUM sequence at the rDNA locus and nicks the bottom-strand DNA in the RASIN site. Second, R2-RT synthesizes the first cDNA strand using the 3’ end of the cleaved DNA as a primer. The 3’ UTR of the template RNA unfolds and dissociates from the R2 protein via reverse transcription. Third, the interaction with 5’ RNA promotes cleavage of the top DNA strand. Second-strand cDNA synthesis is then initiated. Finally, the integration reaction is completed after DNA repair. RUM, retrotransposon upstream motif; RASIN, retrotransposon-associated insertion site; cDNA, complementary DNA.

After first-strand cDNA synthesis, the R2 protein initiates cleavage of the upstream DNA strand [27](Figure 2). It is important to note that the cleavage of the upstream strand occurs sequentially after the cleavage of the downstream strand. According to a previous model of R2 retrotransposition, two R2 protein-RNA complexes bind to upstream and downstream DNA strands, respectively. The 5’ RNA and 3’ UTR regulate the integration reaction of these two complexes. In the presence of 3’ UTR, the R2 protein exhibits enhanced binding efficiency to upstream DNA, effectively initiating the TPRT reaction [27]. Nevertheless, the complex bound to the upstream strand remains in an inhibited state until reverse transcription removes the 3’ UTR region associated with this complex [27] (Figure 2). Once first-strand cDNA synthesis is complete, the R2 protein cleaves the upstream strand. Subsequently, second-strand cDNA synthesis is initiated. Finally, the integration reaction is completed after DNA repair (Figure 2). R2-RT showed higher processivity on ssDNA templates than on RNA templates *in vitro* [24], and a recent study observed second-strand cDNA synthesis by R2-RT *in vitro*, demonstrating that the R2 protein is directly involved in second-strand cDNA synthesis [43]. However, whether host factors contribute to this step and, if so, how they interact with the R2 system remains an important question to be explored.

Although there have been many studies on the integration mechanism of R2 retrotransposons, current research models are almost exclusively based on the D-clade R2Bm protein, which may not be fully applicable to R2 proteins from other clades. For instance, the A-clade R2 protein contains two additional ZFs compared with R2Bm and exhibits significant differences in the mechanisms of DNA recognition. The A-clade R2 protein does not target both the upstream and downstream regions of the rDNA integration site like the D-clade R2, but rather targets only the upstream region [17,44]. The structural evidence of A-clade R2 provides new insights into the basis for template selectivity in A-clade R2 retrotransposons and new nucleic acid recognition mechanisms [45]. It is important to explore more mechanistic differences among different subclade R2 elements, which will help us better understand how the integration specificity of R2 retrotransposons is maintained in different subclade R2 elements.

## Utilization of R2 retrotransposons

### Applications

As typical mobile elements, transposons and retrotransposons can ‘jump’ within a genome. This characteristic is beneficial for the development of new gene integration tools, including transposon-based platforms for CAR-T cell therapy [46–48] and high-throughput mutagenesis library construction for functional genomics research [49]. However, most of the widely used transposon tools integrate randomly in the genome. The progress in converting randomly integrating DNA transposons (such as *PiggyBac* [50,51] and *Sleeping Beauty* [52,53]) or non-specific retrotransposons (such as LINE1 [54]) into site-specific integration tools remains limited. In this context, the R2 retrotransposon specifically targets the multicopy rDNA locus [55], which is located away from the coding regions and serves as a safe harbour site [56–59]. Furthermore, the R2 retrotransposon utilizes RNA intermediates to transfer its own genetic information, and the R2 protein can also be encoded by mRNA, thereby enabling all-RNA-based delivery for gene integration.

Widely used targeted gene integration tools predominantly rely on DNA donors, such as nuclease-mediated homologous recombination [60] or non-homologous end joining [61], and site-specific integrase assisted by prime-editing [62,63]. Compared with these technologies, the RNA-based retrotransposon system provides an attractive option, as the RNA donor can have reduced immunogenicity by chemical nucleoside modifications [64], can be efficiently delivered by non-viral vehicles [65], and can be rapidly degraded in cells [66] with a lower risk of random transgene integration. These advantages make the R2 retrotransposon a promising candidate for developing novel gene addition tools.

**Figure 3.**
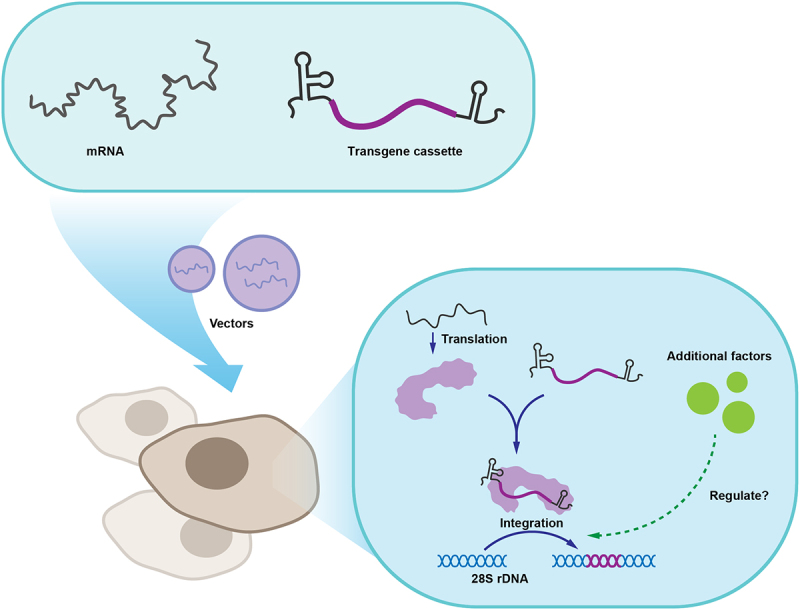
Application of R2 retrotransposons for gene integration.

In 2019, the R2Ol retrotransposon from medaka (*Oryzias latipes*) was used to achieve sequence-specific integration in zebrafish [35]. R2Ol demonstrated highly efficient EGFP integration (up to 95%) in zebrafish germ cells with germline transmissions. However, the initial attempts to integrate transgenes into mammalian cells are still in the early stages [67]. By 2024, two teams systematically investigated the properties of R2 retrotransposons and achieved all-RNA-mediated full-length targeted gene integration in mammalian cells using the R2 retrotransposon (Figure 3). Collins et al. described an approach named precise RNA-mediated insertion of transgenes (PRINT), which can achieve the insertion of an approximately 2 kb transgene into the site-specific rDNA loci with over 50% efficiency in the human hTERT RPE-1 cell line [68]. Our lab developed an engineered R2 tool, named the en-R2Tg system, which enabled effective integration into multiple cell types (approximately 25% efficiency in Huh7 cells and 60% efficiency in mouse embryos) [12]. Some non-LTR retrotransposons, such as LINE1, exhibit higher integration efficiency in *cis* configuration than *trans* complementation because of their *cis* preference [69,70]. To prevent the insertion of the extra DNA sequences of R2 protein into the target locus, the PRINT and en-R2Tg systems both used *trans* constructs, expressing the R2 protein and the RNA donor, respectively, through two RNAs (Figure 3). The transgene is loaded by an RNA donor, which contains R2-specific 5’ UTR and 3’ UTR sequences [12,68,71].

Schematic diagram of the targeted gene integration process using R2 retrotransposons via all-RNA delivery.

With its advantages in terms of safety and deliverability as an all-RNA system, R2 retrotransposons hold great potential for both fundamental biology and therapeutics if developed into an efficient gene addition tool. For example, many monogenic diseases exhibit phenotypic variability and may be caused by more than one specific mutation [72,73]. R2 tools can provide a universal strategy to specifically integrate a complete functional gene into the genome of patients with a disease. Another application of R2 tools is CAR-T cell therapy. Current *ex vivo* CAR-T therapy faces challenges, including difficult manufacturing processes, risks of surgical operation, and high costs [74]. In this situation, *in vivo* direct CAR-T generation is an emerging strategy that can now be achieved through random integration of the payload by lentiviral vectors [75]. R2 tools can provide a more attractive *in vivo* CAR-T strategy because they can be delivered by non-viral vectors and achieve targeted DNA integration.

### Future directions for optimization

In order to promote the practical application of R2 retrotransposons in the future, several issues need to be addressed. One of the most important aspects is to further improve the gene integration efficiency of the R2 tools in mammalian systems. Three points should be considered. First, the integration mechanism of R2 retrotransposons requires further elucidation, such as the details of second-strand cDNA synthesis [43] and the interaction with endogenous cellular factors [34]. Emerging technologies, including cryo-electron microscopy [76] and AI-driven protein structure prediction (e.g. AlphaFold 3) [77] can provide molecular insight into these processes, enabling rational engineering of R2 retrotransposons with enhanced precision and efficiency. Second, when the R2 retrotransposon is applied in mammalian cells, it is crucial to assess whether any accessory components are lacking. For example, in the CRISPR-associated transposases (CASTs) system, ribosomal protein S15, a bacterial host factor, is required for efficient transposition. Adding this bacterial host protein can enhance the integration of CASTs into human cells [78]. Investigating similar accessory component requirements for the R2 system may further improve its efficiency (Figure 3). Third, optimizing RNA stability and delivery strategies can also enhance the all-RNA-mediated gene integration activity of R2 tools because efficient delivery of RNA products with improved stability can ensure an adequate amount of functional RNA within cells.

Furthermore, long-term expression stability of transgenes integrated at rDNA loci requires systematic validation over a long period in various cell types, because it was observed that high insertion copy numbers can reduce the expression intensity of transgenes in human cells [68]. Therefore, improving the programmability of R2 retrotransposons would significantly expand their potential applications. Two approaches may endow R2 retrotransposons with programmability. The first potential strategy involves linking nCas9 or dCas9 with the R2 protein. *In vitro* experiments have demonstrated that R2Bm can be retargeted to other DNA sites by leveraging the programmability of Cas9 [26]. A recent study has confirmed the possibility of reprogramming R2-mediated gene integration in mammalian cells [79], providing novel insights for fully unleashing the activity of R2 retrotransposons. Another strategy is to engineer or replace the DNA-binding region of R2 retrotransposons, as it is crucial for the specificity of DNA recognition. Previous studies have demonstrated that R9Av targets a site distinct from the canonical R2 integration locus, which is likely due to its altered N-terminal domain [16]. Using dedicated protein design strategies, we believe that it is possible to reshape the targeting of R2 retrotransposons.

## Concluding remarks

R2 retrotransposons, which belong to a distinct class of mobile elements, use RNA intermediates for specific integration into rDNA loci via the TPRT mechanism. They enable precise gene integration and all-RNA delivery, thereby providing an expandable biotechnological platform for developing novel therapeutics. We believe that with a deeper understanding of the biology of R2 retrotransposons in the future, highly efficient, programmable, and all-RNA-based gene integration tools can be engineered by utilizing this unique mobile element.

## Data Availability

This study does not generate new data.
